# FightHPV: Design and Evaluation of a Mobile Game to Raise Awareness About Human Papillomavirus and Nudge People to Take Action Against Cervical Cancer

**DOI:** 10.2196/games.8540

**Published:** 2019-04-08

**Authors:** Tomás Ruiz-López, Sagar Sen, Elisabeth Jakobsen, Ameli Tropé, Philip E Castle, Bo Terning Hansen, Mari Nygård

**Affiliations:** 1 HPV Research Group Department of Research Cancer Registry of Norway Oslo Norway; 2 Simula Research Laboratory Oslo Norway; 3 Cancer Registry of Norway Oslo Norway; 4 Albert Einstein College of Medicine New York, NY United States

**Keywords:** papillomavirus vaccines, educational technology, uterine cervical neoplasms, papillomavirus infections, primary prevention, secondary prevention, early detection of cancer, mobile applications, health education, learning

## Abstract

**Background:**

Human papillomavirus (HPV) is the most common sexually transmitted infection globally. High-risk HPV types can cause cervical cancer, other anogenital cancer, and oropharyngeal cancer; low-risk HPV types can cause genital warts. Cervical cancer is highly preventable through HPV vaccination and screening; however, a lack of awareness and knowledge of HPV and these preventive strategies represents an important barrier to reducing the burden of the disease. The rapid development and widespread use of mobile technologies in the last few years present an opportunity to overcome this lack of knowledge and create new, effective, and modern health communication strategies.

**Objective:**

This study aimed to describe the development of a mobile app called FightHPV, a game-based learning tool that educates mobile technology users about HPV, the disease risks associated with HPV infection, and existing preventive methods.

**Methods:**

The first version of FightHPV was improved in a design-development-evaluation loop, which incorporated feedback from a beta testing study of 40 participants, a first focus group of 6 participants aged between 40 and 50 years and a second focus group of 23 participants aged between 16 and 18 years. Gameplay data from the beta testing study were collected using Google Analytics (Google), whereas feedback from focus groups was evaluated qualitatively. Of the 29 focus group participants, 26 returned self-administered questionnaires. HPV knowledge before and after playing the game was evaluated in the 22 participants from the second focus group who returned a questionnaire.

**Results:**

FightHPV communicates concepts about HPV, associated diseases and their prevention by representing relationships among 14 characters in 6 episodes of 10 levels each, with each level being represented by a puzzle. Main concepts were reinforced with text explanations. Beta testing revealed that many players either failed or had to retry several times before succeeding at the more difficult levels in the game. It also revealed that players gave up at around level 47 of 60, which prompted the redesign of FightHPV to increase accessibility to all episodes. Focus group discussions led to several improvements in the user experience and dissemination of health information in the game, such as making all episodes available from the beginning of the game and rewriting the information in a more appealing way. Among the 26 focus group participants who returned a questionnaire, all stated that FightHPV is an appealing educational tool, 69% (18/26) reported that they liked the game, and 81% (21/26) stated that the game was challenging. We observed an increase in HPV knowledge after playing the game (*P*=.001).

**Conclusions:**

FightHPV was easy to access, use, and it increased awareness about HPV infection, its consequences, and preventive measures. FightHPV can be used to educate people to take action against HPV and cervical cancer.

## Introduction

### Background

Human papillomavirus (HPV) is the most common sexually transmitted virus globally [1]. There are over 120 HPV genotypes, over 40 of which infect the anogenital epithelium in women and men. Approximately 14 HPV types are referred to as high-risk types; these can cause cervical cancer and are responsible for a significant proportion of cancers of the vagina, vulva, penis, anus, and oropharynx [2]. Some low-risk HPV types can cause genital warts [3]. Barrier contraceptives, such as condoms, may partially protect against the transmission of HPV infection [4,5]. Approximately 5% of all cancers worldwide are caused by HPV infection [6]. Cervical cancer is one of the most common female cancers worldwide, with global estimates of approximately 530,000 new cases and 270,000 related deaths in 2012 [7]. Although newly developed prophylactic HPV vaccines represent a primary prevention method that can be used to protect against HPV infection and reduce the future burden of cervical cancer [8], screening has been used in many countries for decades as a secondary prevention method. Consistent, periodical cervical cancer screening by cytology or HPV testing can detect precancerous lesions, and if treated in a timely fashion, the progression of these lesions to invasive cervical cancer can be prevented [9].

In almost all countries with a cervical cancer screening program, there is a segment of population (approximately 20%) that does not attend screening, in which a significant proportion of cervical cancers is diagnosed [10-13]. Although mass media campaigns have been effective in improving screening attendance, they have been shown to only have a temporary effect [14]. Awareness of and adult education regarding disease are some of the most important determinants of participation in health services [15,16]. This emphasizes the need for efficient, sustainable, culturally appropriate, and societally relevant approaches to inform women of the disease risks associated with HPV infection, ways to prevent HPV infection, and recent changes in cervical cancer prevention strategies, especially in younger generations who use different media to get their (health) information.

Mobile technologies have developed rapidly since 2007 [17]. In the United States, 95% of the residents have a cell phone and 77% own a smartphone [18]. People spend more time on smartphones than on desktop computers [19], using smartphones up to 5 hours per day on average [20]. Therefore, smart devices appear to be a suitable platform to communicate information to target audiences. Apps for mobile technologies, or *mobile apps*, represent a very promising, burgeoning market and medium to disseminate interventions aimed at changing health behaviors [21]. Mobile apps have the potential to change the way health care systems communicate with their patients in a positive way and facilitate the delivery of patient-centered health care globally [22,23]. Mobile games are among the most popular mobile apps [24], followed by mobile apps for social media platforms, such as Facebook, Twitter, and Snapchat. Information shared through these platforms can influence how we think and act [25].

Given the popularity of mobile games, the concept of gamification, that is, using the elements of game playing for other purposes, has been launched as a novel and promising tool to improve health communication. The idea is that using game thinking and game mechanics in nongame contexts will help users engage in the learning process and thus facilitate knowledge acquisition, positive health-related attitudes, and positive behavioral changes [21,26,27]. As social media is extensively used by the majority of smartphone users, making game aspects shareable through social networks can influence how others think and act. This is called social nudging via gamification, which helps others stick to health-promoting behavior [28]. Social nudging is rooted in the very nature of human behavior, as we are actively involved in the lives of friends and relatives when they discuss lifestyle choices.

### Objectives

In this paper, we describe the principles and processes preceding the launch of a digital game–based learning tool for mobile devices, FightHPV, which aims to communicate concepts that are relevant to understanding the principles behind the sophisticated technology currently used to prevent HPV-related diseases such as cervical cancer.

## Methods

FightHPV was developed in a stepwise manner, applying an iterative design methodology [29]. We originally intended to target only women of cervical cancer screening age, that is, between 25 and 69 years, in Norway. However, as FightHPV aims to leverage the power of mobile communication and social nudging by allowing players to share information about HPV and cervical cancer with their social network, we expanded the target group to include adolescent boys and girls to inform them about HPV vaccination and other preventive methods.

A multidisciplinary team of software engineers, graphic designers, and medical professionals created a skeleton for gamifying selected story lines or narratives with connected text messages to convey health education. The team included researchers in the field of cancer, HPV infection and the management of the Norwegian Cervical Cancer Screening Program. On the basis of extensive domain knowledge, the team decided on a set of characters, gaming rules, game mechanics, and alternatives for social networking. For the programming, Swift for iOS (Apple Inc) and Java (Oracle) for Android (Google) were used. Crash reporting tools from Fabric (Google) were bundled with FightHPV so that the team could receive information on possible malfunctions during the execution of the program; this allowed us to identify and correct software bugs before public distribution. FightHPV was then evaluated by beta testing and 2 focus groups.

The beta testing study included 40 employees of the Cancer Registry of Norway, who were external to the present project but were knowledgeable about cancer. Focus group 1 comprised 6 women aged between 40 and 60 years, who were members of the Norwegian Women’s Public Health Association. Focus group 2 comprised 23 high school students (10 girls and 13 boys, aged between 16 and 18 years) living in Oslo, Norway (Table 1).

Participants in the beta test were invited to download a private (nonaccessible to the general public) release of FightHPV for Android through the testing facilities provided by Google Play. They were encouraged to advance as far as they could in the game and report any flaws they encountered. After beta testing, summary data were collected from Google Analytics for quantitative descriptive analytics, which captured different actions performed by players, including wins, fails, and retries for each level. Following approval from the Data Protection Agency, improved versions of FightHPV were made available through Google Play for Android and TestFlight for iOS.

Participants of focus group 1 downloaded version 2 of FightHPV to their mobile devices, 1 week before attending the focus group discussion held in Oslo, Norway. Participants of focus group 2 downloaded version 3 of FightHPV on their mobile devices and played the game for 2 hours before the focus group discussion held in the participants’ school. Focus group 2 was selected to evaluate how a generation that is used to playing mobile games would respond to a game that teaches lessons about the generation’s own health and its willingness to share the information learned with others who may find it relevant. Although the focus of this research project is cervical cancer, we included boys as they can benefit from vaccination both directly and indirectly [30] and can nudge others to adopt a healthier lifestyle on the basis of the knowledge they acquire from playing FightHPV.

Focus group discussions lasted 45 to 60 mins, were semistructured and led by members of the research team, and were audiotaped at the consent of the participants. For focus group 2, girls and boys had separate sessions, moderated by a female and male member of the research team, respectively. The goal of these discussion sessions was to receive both general and specific feedback on FightHPV. A predetermined set of questions about knowledge and attitudes on HPV and sexual health and how information regarding sexual health can be conveyed to the public was used as a guide. At the start of each focus group discussion, participants were encouraged to freely discuss their experiences with FightHPV, addressing the usefulness of a mobile game to disseminate information about health and their willingness to share this information with others.

Of the 29 individuals who participated in focus group discussions, 26 (4 from focus group 1 and 22 from focus group 2) returned a questionnaire about the game and a test of their knowledge of HPV before and after playing the game. The data from 22 players from focus group 2 were analyzed and tested for statistical significance using the nonparametric Wilcoxon signed rank test. The tests were 1-sided, with null hypotheses of no difference in scores before and after, against alternative hypotheses of a higher score after playing FightHPV.

FightHPV is part of a project approved by the Norwegian Regional Ethical Committee (REK 2015/1926) and the Institutional Data Protection Officer. A liability and copyright terms and conditions of the game, along with logos from Oslo University Hospital and the Cancer Registry of Norway, have been incorporated into the app to enhance players’ trust in the content.

**Table 1:** Characteristics of participants in beta testing and focus groups.

User group	Participants, n	Age range (years)	Gender	Description
Beta test	40	30-60	Male and female	Employees of the Cancer Registry of Norway
Focus group 1	6	40-60	Female	Members of the Norwegian Women's Public Health Association
Focus group 2	23 (10 girls, 13 boys)	16-18	Male and female	High school students

## Results

### Description of FightHPV Game Mechanics, Characters, Game Rules, and Story Lines

**Figure 1:**
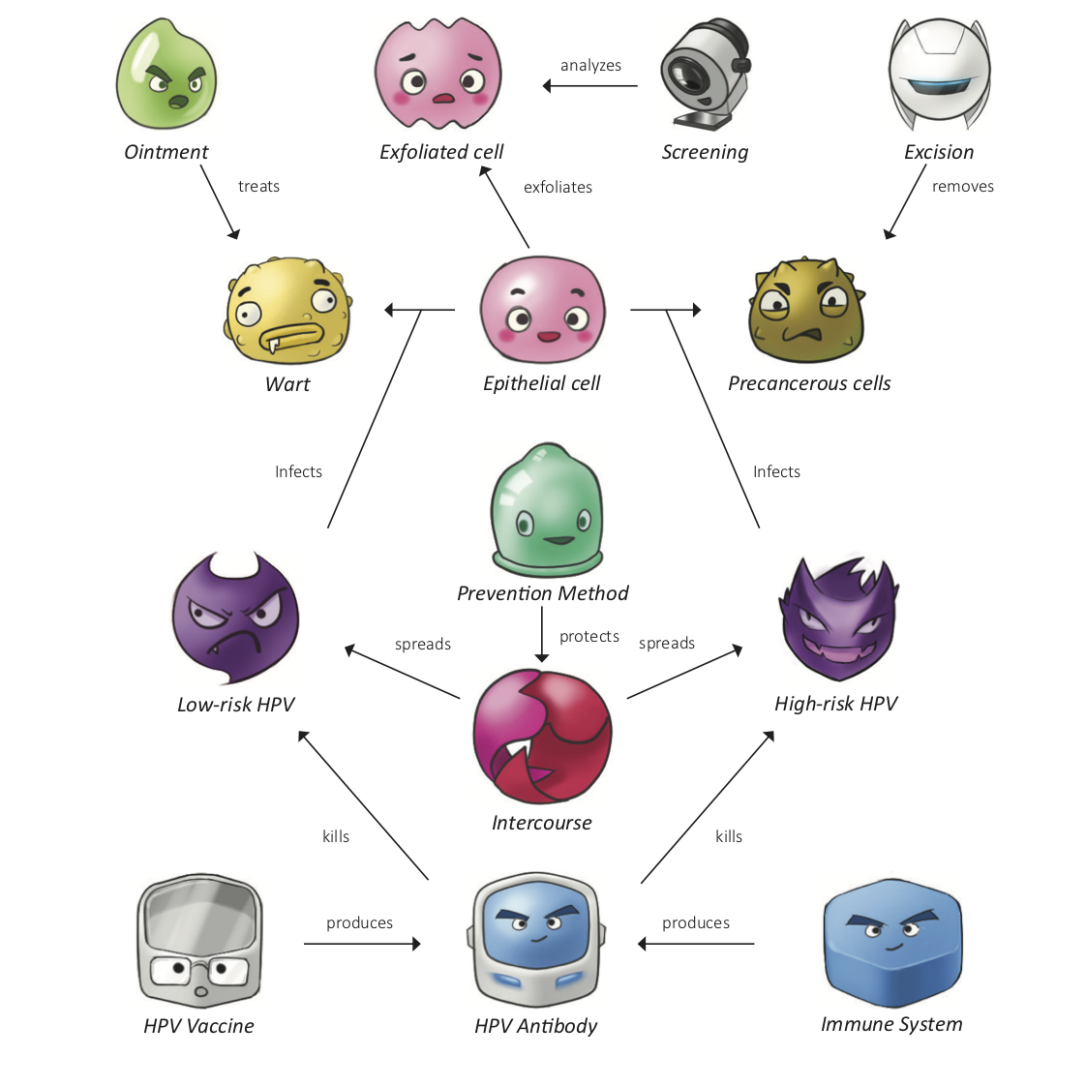
Characters and interactions between characters, that is, game rules in FightHPV. *Low-risk HPV* (human papillomavirus) can transform *Epithelial cell* to *Wart*. *Wart* can be transformed back to *Epithelial cell* using *Ointment*. *High-risk HPV* can transform *Epithelial cell* to *Precancerous cell*. *Precancerous cell* can be transformed by an *Excision* back to *Epithelial cell*. *Intercourse* will release both types of viruses onto the board, but this can be averted by applying *Prevention Method*. *Immune System* in response to *HPV Vaccine* transforms into a powerful *HPV Antibody*, which can kill both *Low-risk HPV* and *High-risk HPV*. Finally, *Exfoliated cells* can be tested by *Screening*.

To mimic the real-world relationships between HPV and the human body, FightHPV includes a set of characters with game rules that define their interactions. The characters (in italics) *Epithelial cell*, *Low-risk HPV*, *High-risk HPV*, *Wart*, *Ointment*, *Precancerous cells*, *Excision*, *Intercourse*, *Prevention Method*, *HPV Vaccine*, *Immune System*, *HPV Antibody*, *Exfoliated cell*, and *Screening* are displayed on a frame of game board–based puzzles. The main character is *Epithelial cell* and the challenge is to unify all the *Epithelial cells* on the game board by changing their positions swiftly, using as few moves as possible. The other characters interact with each other and with *Epithelial cell* (Figure 1).

At the beginning of each level, the characters appear on concentrically placed hexagons or rings, and the player is instructed to solve the challenge by moving the characters on the game board. Each character has a maximum of 6 surrounding positions, which can be taken by any other character on the game board. Furthermore, 2 characters located next to each other trigger the predefined interaction, which can be either positive or negative for the player to solve the challenge (Multimedia Appendix 1).

We selected, amended, and simplified health messages into 60 puzzles (levels), which were then organized into 6 episodes with separate story lines, containing 10 levels each. Information is communicated using relevant characters with predefined rules, which appear in advancing levels within each episode. Gaming difficulty advances gradually from level 1 (simple) to level 10 (more difficult) within each episode. Every new level in the episode either repeats previously introduced information in a more demanding puzzle or introduces a new piece of information.

In episode 1, Epithelial cells, we communicate the role of intact/healthy epithelial tissue and introduce the game mechanics to the player. The actual HPV life cycle is dependent on exploiting the biological process within the epithelial tissue, which covers the outer surfaces of the cervix [31]. Established persistent infection in the epithelium is a necessary trigger for the carcinogenic transformation of epithelial cells. This is a long process that starts with minor cellular abnormalities and advances to more definitive premalignant changes, cancer, and death.

The properties of low- and high-risk HPV to transform normal epithelial cells to warts [3] and precancerous lesions [32], respectively, are presented in episode 2, Low-risk HPV, and episode 3, High-risk HPV. Episode 4, Prevention, presents the spread of both low- and high-risk HPV through unprotected sexual intercourse. The player can use the character *Prevention Measure* (which resembles a condom) to protect *Epithelial cell* against HPV in the game. In episode 5, HPV Vaccine, the principles of vaccination are conveyed by presenting the interplay among the characters *Vaccine*, *Immune System*, *Low-risk HPV*, and *High-risk HPV* [30,33]. Screening principles were gamified in episode 6, Screening.

Communication of health information is reinforced with short messages that appear at the beginning of each puzzle and are related to the goal players have to accomplish. Longer articles that relate to each episode have been written for the mobile app, and links to these articles are available on the puzzle screens for users who want to read more in-depth information.

### In-Game Feedback and Social Networking

**Figure 2:**
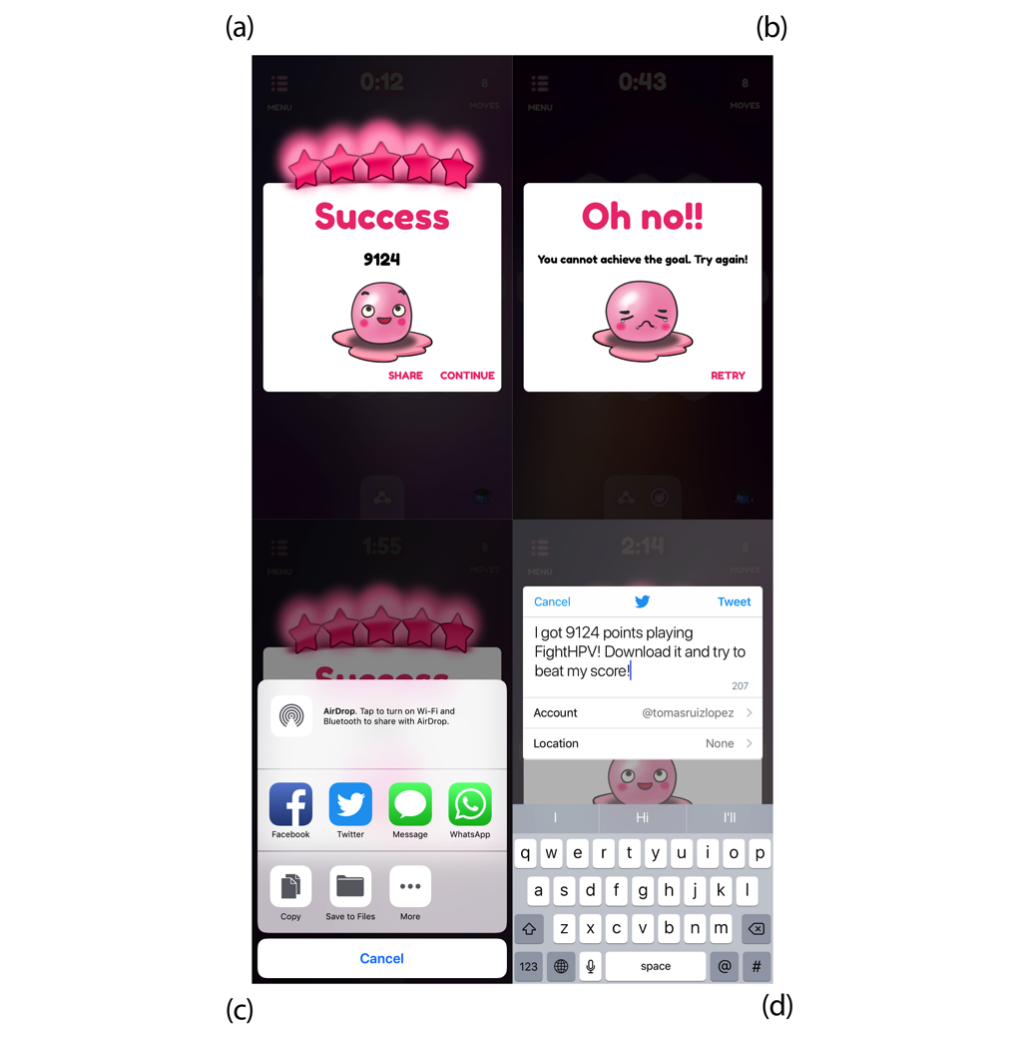
In-game feedback and social networking in FightHPV. Screenshots from immediate feedback to players when they (a) complete or (b) fail a level. Players are encouraged to (c) share their score on social media to drive user acquisition and engagement. An example, (d) shows how users can share their score on Twitter.

Several elements are used to increase players’ motivation to complete the levels, and there are several conditions that determine whether players have successfully completed the levels. Effects and actions in the game are highlighted with sound and character animation to encourage players to retry the level in case of failure. Immediately after completing the level, a score on the scale of 1 to 5 stars with appropriate animation and sound is displayed for the player. The numerical score is calculated on the basis of the remaining number of movements and the time taken to solve the puzzle for that level (Figure 2). Players’ scores are also ranked in leader boards, and players can share their scores and compete with other users. Players can replay a level to find better solutions and increase their score. To further support player engagement and replay, we included achievements. Effects and actions in the game are highlighted with contextual sounds and character animation. For networking, we programmed direct links to all the social network platforms already loaded in the player’s device (Figure 2).

Players with a Norwegian personal identification number, that is, those residing in Norway, have been invited to participate in a separate study to determine the effect of FightHPV on cervical cancer screening attendance (results will be presented in a future article). Once enrollment in that study is finalized, the option to participate in the study will be removed from the FightHPV app.

### Design-Development-Evaluation Loop of FightHPV

**Figure 3:**
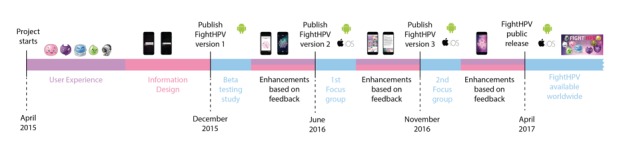
Timeline of the design-development-evaluation loop of FightHPV. Development of FightHPV started in April 2015.

Development of the first version of FightHPV started in April 2015, and beta testing was carried out in December 2015. Version 2 of FightHPV was released and discussed with focus group 1 in June 2016 and version 3 of FightHPV was released and discussed with focus group 2 in November 2016 (Figure 3). Feedback was discussed thoroughly by the team to decide on how to incorporate the suggested changes. Characters, episodes, levels, textual information, in-game communications, and competitive elements were changed in response to the feedback from the focus groups. FightHPV was available to download for free in the App Store and Google Play in Norway in November 2016 and worldwide in April 2017.

Overall, the appearance of all 14 animated characters in version 1 of FightHPV was positively received by participants. They thought that the characters were amusing and cute and that there was a clear distinction between positive and negative characters. In response to the feedback, characters *Intercourse* and *Prevention Method* were slightly changed. Game mechanics, that is, how to move the characters on the game board, was intuitively understood by participants. However, the instruction session on how to solve puzzles was made more explicit and easily available throughout the game (Table 2).

**Table 2:** Summary of the changes in the design of FightHPV after each evaluation. Character names are in italics.

Feature	Initial design	After beta testing	After focus groups 1	After focus groups 2
Characters	14 characters were created	Appearance of *Intercourse* and *Protection Method* needed to be redesigned	—^a^	—
Episodes	60 levels were organized into 6 episodes. Each level was accessible sequentially, that is, only after the previous level was completed	—	Open access to all 6 episodes at any time. However, the 10 levels within the episode were accessible sequentially	—
Levels	Initial puzzle design	Adjusted difficulty for levels with highest proportion of failures	—	Adjusted difficulty for levels with highest proportion of failures
Textual information	Initial writing of human papillomavirus -related information	—	Reduce childlike tone to appeal to more mature audiences	Reduce technical language to increase understandability. Linked real-world images to characters to ease concept comprehension. *Learn more* links from game to longer content articles
In-game feedback	Success or failure only	Background music and sound effects added	—	Animations after game actions added
Competitive elements	Achievements and leader boards	—	Social features added to compare player’s performance with others/friends	—
User interface miscellaneous	—	—	Ability to toggle music/sound on and off	Changed text font for readability

^a^No changes were made to this feature at this development stage.

**Figure 4:**
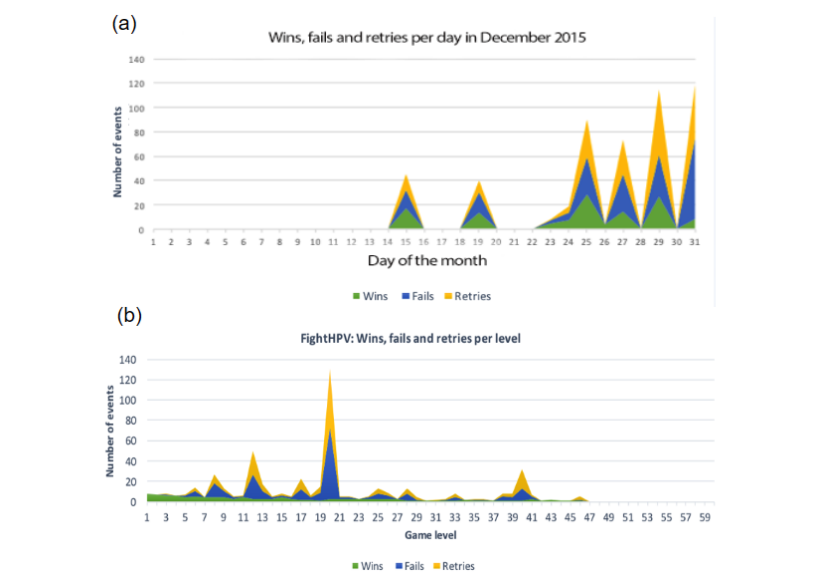
Results from beta testing. (a) Frequency of wins, fails, and retries per day during a period of 1 month (December 2015). (b) Frequency of wins, fails, and retries per level. HPV: human papillomavirus.

All levels were accessible sequentially in version 1, meaning that the player had to complete all the levels in episode 1 before advancing to episode 2, and so forth. Data from Google Analytics (Table 3 and Figure 4) showed that episode 2 was played 450 times and was the most frequently played episode. Several peaks in failures and retries were shown for levels 5, 8, 12, 20, and 40, indicating that those levels were considerably more difficult than others and the players were more likely not to complete them. The frequency of actions after level 50 was negligible, and only 1 player out of 40 completed the whole game. During beta testing, the game was played most frequently during the weekend following its release (December 14-16, 2015), immediately after downloading, and during the holiday season (December 23-31, 2015). Assessing how players navigated among different sections of the app revealed that 13% (5/40) of participants read the longer articles, whereas all participants played the game by solving puzzles.

The feedback from beta testing and from Google Analytics demonstrated that players stopped playing the game if the level was too difficult, and without completing the level, there was no way around to advance to the next episode. We therefore simplified levels that were causing the most trouble and allowed direct access to every episode from the beginning of the game.

**Table 3:** Results from beta testing. Frequency of in-game events.

Episode	Game actions	Event count
1. Epithelial cells	Connecting cells on the board	307
2. Low-risk HPV^a^	Connecting cells on the board	459
2. Low-risk HPV	Using *Ointment*	354
3. High-risk infection	Connecting cells on the board	96
4. Prevention	Using *Prevention Method*	73
5. HPV vaccine	Generating antibody	27
5. HPV vaccine	Eliminating virus	72
6. Screening	Using *Exfoliated cell*	15
6. Screening	Using *Excision*	15

^a^HPV: human papillomavirus.

Textual information presented in the game, which reinforced the health message, was considered useful by all participants of the beta test group and focus groups. However, the first focus group felt that the tone of these messages was either too childish or too complicated. Longer articles, in contrast, were perceived as too technical. Short messages, longer articles, and the text that described the medical concepts early in the game were rephrased by the writer of the development team. The text font for the entire game was changed to improve readability (Table 2).

Upon user request, we added sound effects, background music, and animations to accompany important events, which enhanced feedback for the actions performed by the player during the game. We established a visual relationship among the characters, real images in the *Learn more* section, and allowed players to turn music and sound effects on and off. Furthermore, sharing on social media was incorporated into leader boards and achievements to drive user engagement (Table 2).

### General Feedback on FightHPV From Focus Groups and Effect on Knowledge

The overall feedback from the focus groups was positive; out of the 26 participants who returned their questionnaire, all stated that FightHPV is an appealing educational tool, 69% (18/26) reported that they liked the game, and 81% (21/26) stated that the game was challenging. Participants from focus group 2 had little a priori knowledge about HPV and HPV-related diseases, whereas participants from focus group 1 reported medium to high knowledge. During the discussion session, participants in focus group 2 specifically raised questions about HPV and how it can affect their health. They found the game to be thought provoking and requested more information on HPV. The girls’ group suggested that this type of health information should be mandatory in schools, and they considered information about HPV to be a relevant topic for their well-being. Of the 22 participants in focus group 2 who returned a questionnaire, all showed a statistically significant improvement in their knowledge about epithelial cells, HPV, and HPV transmission, with a median and interquartile range (IQR) that ranged from very low to low before playing FightHPV, to low and medium after playing FightHPV. Concepts about HPV vaccination were best understood, with the highest IQR (Table 4). All participants from focus group 2 were willing to share this information within their close social circles as they thought it would be beneficial for them.

**Table 4:** Knowledge about different topics related to human papillomavirus before and after playing FightHPV. Self-reported knowledge scores ranging from 1 (very low) to 5 (very high) among 22 participants of focus group 2 who returned the questionnaire.

Topics, scores		Scale					Median score (interquartile range)	*P* value (before vs after)^a^
		1	2	3	4	5		
**Epithelial cells**								.001
	Before	14	4	4	0	0	1.00 (1.00- 2.00)	—^b^	
	After	3	9	8	2	0	2.00 (2.00-3.00)	—	
**HPV^c^**								.001
	Before	13	4	4	1	0	1.00 (1.00-2.00)	—	
	After	5	7	6	3	1	2.00 (2.00-3.00)	—	
**HPV vaccine**								.006
	Before	9	8	3	2	0	2.00 (1.00-2.00)	—	
	After	3	9	4	5	1	2.00 (2.00-3.75)	—	
**HPV transmission**								.006
	Before	11	6	4	0	1	1.50 (1.00-2.00)	—	
	After	5	9	3	2	3	2.00 (2.00-3.00)	—	
**Screening**								.01
	Before	16	3	2	1	0	1.00 (1.00-1.75)	—	
	After	10	8	2	1	1	2.00 (1.00-2.00)	—	

^a^One-sided Wilcoxon signed rank test (N=22; 10 females, 12 males, all attending Ullern High School).

^b^Not applicable.

^c^HPV: human papillomavirus.

## Discussion

### Principal Findings

To the best of our knowledge, FightHPV is the first game to be released globally that gamifies health information about HPV and cervical cancer prevention. Participants in our user groups reacted positively toward educational game, and we were able to demonstrate a statistically significant positive effect of FightHPV on knowledge. In addition, focus group discussions revealed a significant lack of knowledge regarding HPV before playing the game, confirming the need for enhanced communication regarding HPV to the population. Furthermore, FightHPV was designed to create a network for knowledge sharing among people by employing social media, which has become integrated in our daily lives. Gamification and nudging have already been explored in other domains, such as fitness apps, with some success, which suggests this approach could be equally beneficial in communicating serious health information, raising awareness, and eliminating misconceptions about HPV.

Use of FightHPV can influence behaviors that are relevant to individual decisions regarding whether to receive HPV vaccination or attend cervical cancer screening. Social cognitive theories and empirical studies suggest that directed educational interventions that influence attitude, knowledge, and motivation are most effective when accounting for the context in which the targeted health behaviors take place [34]. As social norms shaped by social media had a devastating effect on HPV vaccination in Japan and Denmark [35,36], we wanted FightHPV to be easily shared in social circles, not only to engage more people in the game but also to share correct information and send a reminder about the importance of screening to family and friends.

To improve individual learning, we incorporated the following features into FightHPV, which are typical in digital game–based learning [37]: (1) a set of rules and constraints that described relationships and effects among characters, (2) several dynamic responses to players’ actions, for example, sound, scoring, animations of effects, and characters, which communicate if a player made a right or wrong move; (3) direct access to episodes with different levels of difficulty, enabling learners to experience a feeling of self-efficacy, (4) gradual advancement in learning outcome within an episode. The combination of amusing characters with animation was seen as entertaining and increased player willingness to repeat levels and enhance cognitive changes, which is an important characteristic of digital game–based learning. To enhance player/learner motivation and engagement in acquiring new knowledge, we created story lines that would most help the layperson to understand virus transmission and what actions can be taken to avoid viral infection and cancer progression. We drew a parallel between the relationships among HPV concepts and typical video game quests, for example, the enemies—*Low-risk HPV* and *High-risk HPV* —trying to threaten other characters, that is, the *Epithelial cell*, and a hero, that is, the player, who needs to use tools (ie, the characters *HPV Vaccine* and *Screening*) to defeat the enemy. Visually appealing characters with interactive animations were created to further increase players’ motivation to advance in or to repeat the game.

Beliefs that HPV infection and cervical cancer are not real threats to an individual’s health and fatalistic beliefs that nothing can be done to reduce one’s risk of dying from cancer have been reported as barriers that prevent women from attending screening and/or accepting to be vaccinated against HPV [37,38]. Interactive animation of characters according to player’s moves and gaming rules created settings, which bridged the gap between abstract concepts of the natural history of cancer and a person’s own body and helped to personalize the information. For example, the *Intercourse* character illustrates the main route of HPV transmission among humans. A character, *Prevention Method*, was also introduced, which helps the player get protected against HPV released from *Intercourse*. As a result of how these characters behave in the game, the puzzles featuring them become harder, depending on how long it takes to use a *Prevention Method*. This helps to communicate the importance of prevention. The episode *HPV Vaccination* presents 3 characters: (1) the *Immune System*, which is not able to fight HPV by itself, (2) the *HPV Vaccine*, which gives powers to the *Immune System* to fight HPV, and (3) the *HPV Antibody*, which appears after *HPV Vaccine* and has the ability to clean the board of HPVs.

We acknowledge that the applied interaction between characters and rules represents a considerable simplification of the real-life situation. On the other hand, simplification allows us to communicate a few take-home messages and address the principle of the increasing difficulty of levels within the episodes to provide a good gaming experience for player.

Appropriate instructions have been shown to help learners target relevant information and affect cognitive processing [38]. However, instructions given in the context of a digital-based learning game are thought to be playful and not focus on educational goals, thus eliciting shallower cognitive processing. To improve learning effectiveness, we presented textual information as well, either as short sentences or long articles, to influence deeper cognitive processes. Short sentences appeared at the beginning of each level and were purposely designed to be in a dialogue form, as if the characters in the game were telling a story. In this way, players can relate the text they read to the puzzle they are solving and vice versa. Although text information is easily bypassed for a better gaming experience, players can solve puzzles more easily if they pay attention to the text information. Furthermore, focus group discussions showed that FightHPV provoked players to think about their own behavior related to HPV and raise questions such as, “When was the last time I attended screening?” “Did I have unprotected sexual contact?” and “Have I been exposed to the virus?” In this way, players were able to personalize the information and relate their own life experiences to what they were learning.

FightHPV was also designed to trigger the desire to do something for others: “Should I vaccinate my children?” “Does my mother, sister, wife, daughter, friend, know how frequently they should go to screening?” Players are encouraged to share information with their relatives and acquaintances so that, even though they may not ever play, they can receive the information that we want to transmit. It is somewhat surprising that social nudging has not been used more actively in intervention trials to influence behavior; however, it is gaining attention [34].

Although HPV-related health messages are well suited for gaming, it took us approximately 1.5 years to develop FightHPV, and we encountered several challenges. The design-development-evaluation loop proved to be a useful method to develop different aspects of the game. At the start, the results from beta testing reassured us that using puzzles in a board game format engaged the players and that the mechanics of the game were simple and easy to learn, which made the game intuitive and easily approachable by almost all age groups without any previous gaming knowledge. However, along with being simple, the mechanics should also provide an adequate challenge to keep players engaged. To avoid user frustration at the early stages of the game, or boredom and lack of challenge at the end, it must have an increasing level of difficulty [39]. In the very early stages of development, we discovered shortcomings in the progression of difficulty in the game by using Google Analytics. Objective, numerical data feedback on failures, and retries on specific levels were essential to understand which part of the game was too difficult. To fine-tune the progression of the difficulty levels, we proposed the use of computational methods to decide the difficulty level, under the assumption that a level was equally hard to solve for a human and a computer. A recent paper described the formalization of the game board and the characters in a constraint program to solve FightHPV levels [40]. The solution of the constraint program was the sequence of moves to go from the initial state to a final state in which the winning condition is met.

An important part of raising awareness about HPV prevention through FightHPV is to attract as many users as possible and engage them to play through all the levels in the game. In this way, they will receive all the important health messages. Furthermore, receiving information repetitively over several playing sessions will help the user retain this knowledge for the future. Achievements and leader boards are commonly used game elements that increase user engagement. In addition to completing the levels in FightHPV, we created 44 side missions that users can complete by playing levels multiple times. Each time players complete one of these missions, they get a badge acknowledging their achievements. Global leader boards were created on the basis of the sum of players’ scores in each level. Replaying some levels can help players get better scores and rank higher on the leader board. As FightHPV has been designed for the mobile platforms Android and iOS, we used Google Play Games and Game Center, respectively, to implement these features. They also include some social features so that players can compare their results with friends’ results and challenge them, thus increasing user acquisition and engagement.

Although FightHPV was developed in the Norwegian context, it is technically simple to expand or modify the content of the game. The organization of the information into episodes allows developers to introduce new topics with minimal technical effort. For example, in Africa, it might be relevant to add an episode to communicate the interaction between HIV and HPV. Translation of the content to other languages is technically simple; however, we suggest that each country should evaluate the local context and communicate correct guidelines for vaccination and cervical cancer screening. Furthermore, short textual messages can be redesigned into pictograms to reach a wider audience. Hence, FightHPV can be adapted for use in different cultural and medical contexts.

### Limitations

This paper describes our experiences in creating the very first game-based learning tool to convey simplified health information on HPV to a layperson. A few, if any, textbooks were available to provide guidance on how to design such a tool or to provide information on the pros and cons of using different development methods. We strongly feel that the iterative design, which has been widely applied in software engineering for agility and realizing a good product or market fit, was an optimal choice. Arguably, enhanced size and compositions of the evaluation groups could have improved the variety of the feedback; however, we were able to evaluate and redesign the app on the basis of almost all of the relevant suggestions provided by the user groups. The development team might have benefited from incorporating aspects of cognitive psychology in the evaluation of the feedback from the user groups. However, we feel that the methodology used was sufficiently rigorous to conform to a traditional systematic approach. Employees of the Cancer Registry of Norway participated in the beta test and most of these people are familiar with the concepts surrounding HPV and cervical cancer. They essentially tested only the playability of the game, without paying much attention to the medical content. This was observable because of the low number of visits to the information section of the app (Google Analytics, data not shown).

Focus group 1 had a small sample size (6 women), and although the women’s qualitative feedback led to several game adjustments, it is not possible to suggest that these modifications would have appealed to the entire intended target group. Focus group 2 comprised high school students who were not of screening age, which is between 25 and 69 years in Norway. Although the game and the medical information it communicated were of interest to these participants, it is hard to extrapolate the same level of interest to the screening-eligible age group. It was also hard to know if the participants would have the attention span required to learn about every concept communicated in the game. This is primarily because of app fatigue and the availability of a wide range of apps. We did not observe significant barriers to the discussion of sensitive issues, perhaps as girls and boys were interviewed separately. In addition, there were no dominant voices in any of the focus groups to steer the discussion in only one direction. All focus group discussions included people who either had the Android phone/tablet or an iPhone/iPad based on the iOS operating system. The game was not developed for other mobile platforms; however, the market share for other mobile platforms is low in Norway.

### Conclusions

We have successfully designed and released the game-based learning tool FightHPV, which at the time of this study, had been downloaded more than 12,000 times in more than 45 different countries on the Android and iOS platforms. Engineering, biology, medicine, epidemiology, art, and technology interventions were incorporated to create a modern platform to convey relevant information on basic biology and anatomy, which is needed to make informed decisions on personal health. Furthermore, FightHPV also has the possibility to nudge people to take action against cervical cancer. The proposed concept is flexible and scalable, and the content of the game can be expanded to large populations and different cultures. The usage of apps for health purposes is receiving some attention, thanks to initiatives such as Research Kit from Apple, as it enables people to create platforms for the communication and monitoring of different aspects of health. However, more studies are needed to quantify the extent to which these apps and games can actually change health behavior.

## Appendix

Multimedia Appendix 1 Game mechanics of FightHPV.

## Abbreviations

HPV: human papillomavirus
IQR: interquartile range
