# Supplementary material for: FightHPV: Design and Evaluation of a Mobile Game to Raise Awareness About Human Papillomavirus and Nudge People to Take Action Against Cervical Cancer
Source: JMIR Serious Games. 2019 Apr 8;7(2):e8540. doi: 10.2196/games.8540 (PMC6475825; doi:10.2196/games.8540)
Supplement: Multimedia Appendix 1 [file games_v7i2e8540_app1.pdf]

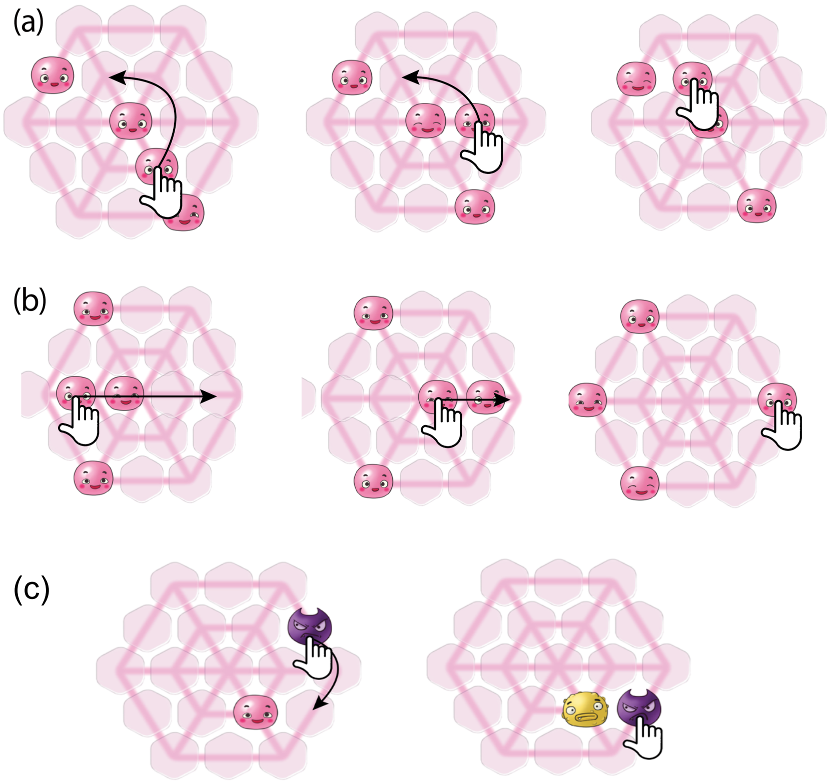

The player can change characters' location on the board by selecting the character and (a) rotating it along the ring of hexagons; the rest of the characters in the rotating ring remain in the same relative position to each other; (b) dragging it to one of the three main diagonals of the hexagonal board. To prevent characters being pushed off the board, the diagonals are circular; when a character goes off the board, it re-enters the diagonal from the opposite side.

Character interactions and their effects appear when the player places characters beside each other (c). Moving *Low-Risk HPV* next to an *Epithelial Cell*, causes the latter to turn into a *Wart.* This effect is determined by the pre-defined game rules, which mimic clinical outcomes following infection with low-risk HPV*.* The game is considered over if the player cannot solve the puzzle, i.e., cannot collect all the *Epithelial Cells* that were present on the board. At next try, the player has learned that s/he must avoid moves that place *Epithelial Cell* and *Low-Risk HPV* in neighbouring positions on the board, in order to complete this puzzle.
